# Spinosin: A Critical Updated Review on Pharmacology, Pharmacokinetics, Toxicity and Translational Bottlenecks

**DOI:** 10.3390/molecules31152567

**Published:** 2026-07-23

**Authors:** Keer Lu, Congyao Wang, Ji Li

**Affiliations:** School of Basic Medical Science, Heilongjiang University of Chinese Medicine, No. 24 Heping Street, Xiangfang District, Harbin 150040, China; 15131165205@163.com (K.L.); wcywcy0113@163.com (C.W.)

**Keywords:** spinosin, pharmacology, pharmacokinetics, drug delivery systems, derivatives, review

## Abstract

Ziziphi Spinosae Semen (ZSS) is a traditional East Asian sedative–hypnotic herb with over 2000 years of clinical application. Spinosin (SPI), a characteristic flavone-C-glycoside, is the official quality marker and principal bioactive constituent of ZSS. Despite extensive research on SPI in recent years, a timely, comprehensive review integrating its pharmacological mechanisms, pharmacokinetic barriers, and translational strategies remains absent. Herein, a systematic literature search was conducted up to 31 May 2026, and we synthesize all available evidence on SPI’s chemical properties, natural sources, pharmacology, pharmacokinetics, toxicology, structural derivatives, and advanced drug delivery systems. Our analysis reveals that SPI exerts broad-spectrum pharmacological activities via multi-target modulation of serotonergic/GABAergic neurotransmission, the ERK/CREB/BDNF axis, and the Nrf2/HO-1 pathway. However, its clinical translation is severely hindered by extremely low oral bioavailability (<1%) and limited blood–brain barrier penetration due to poor aqueous solubility and P-glycoprotein-mediated efflux. Novel formulations have achieved up to 5-fold enhancement in oral bioavailability in preclinical models. While toxicological studies support a favorable safety profile, long-term toxicity and human pharmacokinetic data are lacking. This review critically discusses key translational bottlenecks and proposes evidence-based future directions to advance SPI as a natural neurotherapeutic agent.

## 1. Introduction

Natural products represent an indispensable reservoir of bioactive molecules, and a large number of modern pharmaceuticals have been developed, directly or indirectly, from plant sources [1]. Traditional Chinese Medicine (TCM), with over 2000 years of empirical use, provides a rich repository of bioactive compounds. Among these, Ziziphi Spinosae Semen (ZSS), the dried mature seeds of *Ziziphus jujuba* Mill. var. *spinosa* (Bunge) Hu ex H.F.Chow, is a widely used sedative–hypnotic herb in Asian traditional medical systems, including TCM, Korean Medicine, and Japanese Kampo Medicine. In China, it has a 2000-year history of documented use for insomnia and is classified as a “medicine and food homologous” substance. It is widely distributed across Asia, Europe, and Australia, with main production regions in northwest China and the Yellow River Basin [2]. It is officially recorded in all editions of the Pharmacopeia of the People’s Republic of China and is used clinically to treat insomnia, anxiety, palpitations, and vivid dreaming.

Extensive phytochemical investigations have revealed that ZSS contains a rich array of chemical components, including flavonoids, triterpenoid saponins, alkaloids, fatty acids, and volatile oils [2]. Among them, spinosin (SPI), a characteristic flavone-C-glycoside enriched in ZSS, serves as the official quality-control marker for ZSS in the Pharmacopeia of the People’s Republic of China (2025 edition) and is widely recognized as the principal bioactive component underlying the traditional sedative–hypnotic effect of ZSS. Although SPI has also been identified in a small number of other medicinal plants, including other Ziziphus species, *Passiflora edulis* Sims, and *Acorus calamus* L. var. *angustatus* Besser, its content in these plants is extremely low. There is no traditional ethnopharmacological evidence linking these plants to sedative–hypnotic applications via SPI, making ZSS the only medicinally relevant source of SPI with solid traditional application support [3,4,5]. Beyond its well-established sedative–hypnotic and anxiolytic activities, SPI has recently exhibited neuroprotective, anti-inflammatory, antidepressant, antioxidant, and cardioprotective properties, generating interest in its potential for treating neurological, psychiatric, and other disorders [6].

Despite significant advances in SPI research over the past two decades, several critical knowledge gaps remain unaddressed. First, previous reviews published before 2023 have focused almost exclusively on the sedative–hypnotic effects of SPI, and no comprehensive overview has systematically integrated its expanding pharmacological spectrum (including neuroprotection, hepatoprotection, and cardioprotection), pharmacokinetic limitations, structural derivatives, and novel drug delivery systems. Second, the crosstalk between key signaling pathways mediating SPI’s multi-target effects remains poorly understood, and the relative contribution of SPI metabolites to its in vivo efficacy has not been explored. Third, while recent years have witnessed rapid progress in SPI formulation development, these advances have not been systematically synthesized, and the clinical translation potential of different delivery strategies has not been comparatively evaluated. Finally, all existing pharmacokinetic and toxicological data are derived from animal models, with no human pharmacokinetic studies or long-term safety data available, representing the most significant barrier to clinical development.

To fill these critical knowledge gaps, we conducted a systematic literature search and present the most comprehensive and up-to-date review of SPI research, covering all relevant literature published up to 31 May 2026. In this review, we systematically summarize the chemical properties, pharmacological activities, and underlying molecular mechanisms, pharmacokinetic profiles, safety evaluations, structural derivatives, and novel drug delivery systems of SPI. Furthermore, we critically evaluate the strengths and limitations of existing preclinical studies, discuss the key translational bottlenecks, and propose evidence-based strategies for future research. This review aims to provide a valuable reference for researchers working in natural product pharmacology, pharmaceutical development, and translational medicine, and to facilitate the clinical translation of SPI as a safe and effective natural neurotherapeutic agent.

## 2. Materials and Methods

### 2.1. Search Strategy

This review retrieved literature from PubMed, Web of Science and CNKI, covering all documents from database establishment to 31 May 2026. Boolean operators were adopted to combine keywords related to spinosin with terms involving pharmacology, pharmaceutical development and translational research. The retrieval formula was as follows: (“spinosin” OR “2′′-O-beta-D-glucopyranosyl swertisin”) AND (“pharmacology” OR “pharmacokinetics” OR “toxicity” OR “derivatives” OR “drug delivery systems” OR “biological activity” OR “molecular mechanism” OR “translational potential” OR “clinical application”). There were no restrictions on publication language, publication time or research design. No built-in retrieval filters were used during document retrieval. We did not contact researchers, industry experts or manufacturers to obtain unpublished data or supplementary experimental results.

### 2.2. Inclusion and Exclusion Criteria

The literature satisfying all the following criteria was included in this review: (1) original studies investigating the bioactivity, pharmacological mechanism, pharmacokinetic properties, toxicological characteristics, structural modification derivatives or drug delivery systems of spinosin (SPI); (2) in vitro (cell lines, isolated tissues, molecular binding experiments) or in vivo animal experimental studies; (3) studies that provided clear quantitative data on therapeutic efficacy, molecular targets or pharmaceutical parameters; (4) research taking SPI as the sole active ingredient or main bioactive component with independent quantitative detection and functional evaluation.

Exclusion criteria were set as: (1) review papers, meta-analyses, case reports, conference abstracts and book chapters; (2) compound prescriptions or crude Ziziphi Spinosae Semen extracts without separate quantification and independent functional verification of SPI; (3) duplicate literature and studies with unavailable full texts; (4) papers only establishing SPI detection methods without relevant biological or pharmaceutical experimental data; (5) studies with obvious experimental defects and unrepeatable results.

### 2.3. Study Selection and Data Extraction

Two reviewers (K.L. and C.W.) independently screened titles and abstracts and subsequently assessed the full texts of potentially eligible studies. Discrepancies were resolved by discussion with a third senior reviewer (J.L.). Data were extracted using a standardized form covering study characteristics, experimental models, dosages, key outcomes, and core signaling pathways.

### 2.4. Study Selection Process

A total of 128 articles were initially retrieved. After the removal of duplicates using EndNote X9, 117 unique records remained. During title and abstract screening, 30 records were excluded because they were clearly irrelevant to the review topic. The full texts of the remaining 87 articles were then obtained and assessed against the predefined eligibility criteria. All 87 articles were found to meet the inclusion criteria and were thus included in this narrative review. The entire screening and selection process was conducted through consensus between the two reviewers, with disagreements adjudicated by the third reviewer.

## 3. Chemical Properties and Phytogenic Origin of SPI

### 3.1. Chemical Structure, Physicochemical Properties

**Figure 1:**
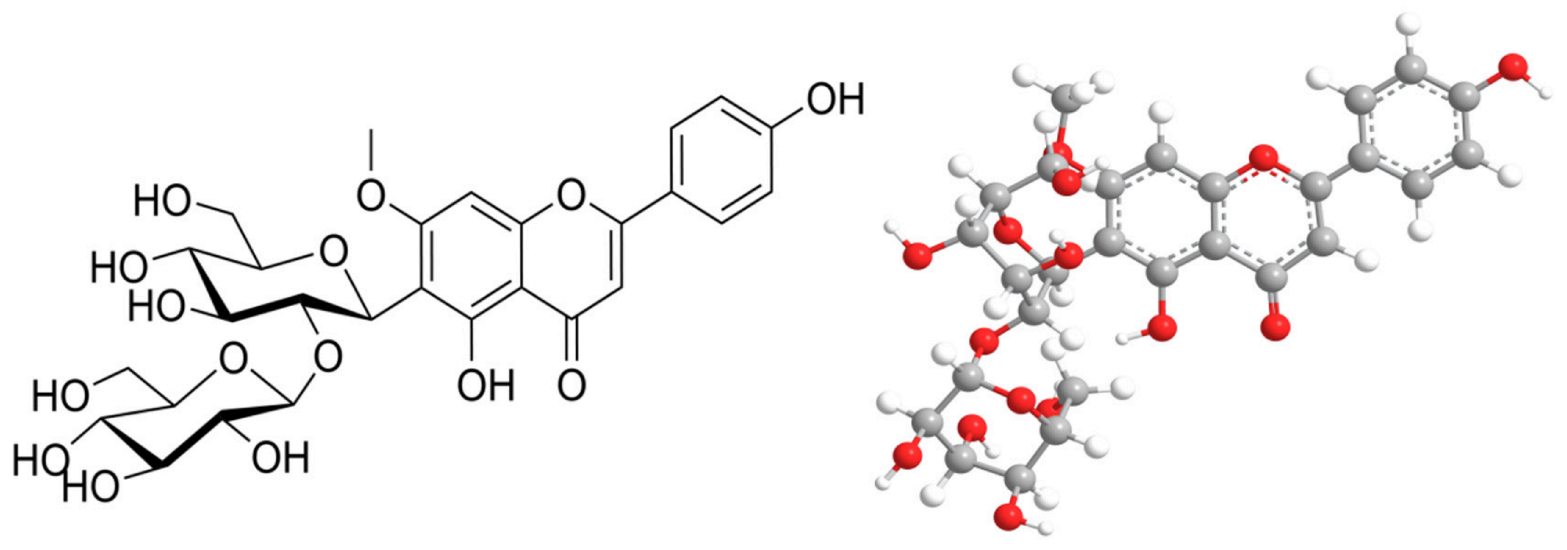
Structure of SPI. (PubChem CID: 471002; (**left**): 2D skeleton; (**right**): 3D ball-and-stick model).

SPI (chemical name: 2′′-O-β-D-glucopyranosyl swertisin, molecular formula: C_28_H_32_O_15_, Figure 1) is a natural flavonoid that mainly exists in dried and ripe seeds of ZSS [7]. Its core structure consists of a flavone aglycone skeleton, with multiple hydroxyl (−OH) groups and one methoxy (−OCH_3_) group substituted on the A-ring, and two glycosyl substituents (glucose moieties) linked to the C-ring and B-ring via stable C-glycosidic bonds. The C-glycosidic bond structure confers SPI high chemical stability under acidic and thermal conditions, while the multi-hydroxyl and glycosyl substituents are the key structural features underlying its biological activity [8]. In its pure form, SPI appears as a pale yellow crystalline powder with a precise molecular weight of 608.55 g/mol. Its key physicochemical properties are systematically summarized in Table 1.

The physicochemical parameters reveal two core characteristics of SPI that directly determine its druggability and pharmaceutical development potential: First, SPI has theoretical high hydrophilicity (consensus Log *p* = −1.07) and a large polar surface area (PSA = 294.2 Å^2^). However, due to its rigid flavone aglycone skeleton, strong intramolecular hydrogen bonding, and tight crystal lattice structure, the experimentally measured aqueous solubility of SPI is extremely low (only ~12 μg/mL at 25 °C) [9]. This paradox directly leads to its core druggability bottlenecks: poor gastrointestinal membrane permeability, extremely low oral bioavailability (less than 1% in rats), and limited blood–brain barrier (BBB) penetration [10,11]. To mitigate these significant limitations, diverse strategies have been pursued, encompassing formulation engineering, structural derivatization, and next-generation drug delivery systems, all of which will be thoroughly examined in the forthcoming sections. Moreover, SPI demonstrates excellent chemical stability under conventional storage, processing, and extraction conditions, with no significant degradation observed during standard heating and reflux extraction [8]. This thermal stability enables the effective use of common processing techniques; specifically, water decoction, stir-frying, and compatibility within herbal formulas have been shown to significantly improve the dissolution rate and in vivo exposure of SPI [12]. Such stability is highly advantageous for industrial manufacturing, ensuring that SPI remains intact during routine production and providing a solid basis for developing stable, reproducible SPI-based formulations.

### 3.2. Extraction, Purification and Biosynthesis of SPI

Given its widespread distribution and crucial pharmacological significance, numerous studies have focused on the extraction and purification of SPI from diverse medicinal materials. Nevertheless, the natural SPI content in medicinal plants is relatively low, which has continuously driven the optimization and improvement in extraction protocols. Highly efficient extraction approaches are essential not only for qualitative and quantitative phytochemical analysis but also for the large-scale preparation and further therapeutic development of SPI.

The extraction of SPI generally follows a multi-step process from crude extraction to purification. For preliminary enrichment, liquid–liquid extraction and macroporous adsorption resin chromatography are the most commonly used methods, with the n-butanol fraction of the crude extract showing the highest SPI enrichment; AB-8, D101, and HPD-100 macroporous resins exhibit the optimal adsorption and desorption effects on SPI, effectively removing impurities such as sugars, pigments, and saponins and increasing SPI purity to over 20% [8,13]. Subsequent targeted separation and purification are typically achieved using silica gel column chromatography, medium-pressure liquid chromatography (MPLC), and polyamide column chromatography, while high-speed countercurrent chromatography (HSCCC) is also employed for SPI separation due to its advantages of no irreversible adsorption, high sample recovery, and continuous large-scale separation [14]. Preparative high-performance liquid chromatography (prep-HPLC) serves as the key technology for obtaining high-purity SPI (purity ≥ 98%), which is widely used in reference substance preparation and laboratory-scale sample production, and the structure and purity of the final product are usually confirmed by thin-layer chromatography (TLC), high-performance liquid chromatography (HPLC), HPLC-mass spectrometry (HPLC-MS), nuclear magnetic resonance (NMR), and electrospray ionization time-of-flight mass spectrometry (ESI-TOF-MS) [15,16,17,18]. At present, SPI extraction and purification still face three core bottlenecks: the extremely low natural content of SPI in ZSS results in a total extraction and purification yield of only 0.02–0.05%, which fails to meet the demand for large-scale pharmaceutical production; existing technologies are associated with high production costs, long production cycles, and significant environmental pollution, failing to meet green production requirements; and the complex C-glycoside structure of SPI makes its total chemical synthesis difficult, with low-yield and high-cost synthetic routes that cannot be industrialized.

In recent years, biosynthesis and synthetic biology-based preparation of SPI have become research hotspots to address these bottlenecks: the biosynthetic pathway of SPI in ZSS has been preliminarily analyzed, and the key enzyme genes involved in flavone aglycone and glycosyl substituent synthesis have been identified, laying a foundation for constructing microbial cell factories for efficient SPI synthesis; in addition, progress has been made in the semi-synthesis of SPI using swertisin as the starting material, providing a new approach for large-scale SPI preparation.

### 3.3. Natural Sources of SPI

SPI is a flavone-C-glycoside compound mainly isolated from ZSS, the dried ripe seeds of *Ziziphus jujuba* Mill. var. *spinosa*, which is the only medicinal source with clear multi-ethnic traditional application and sufficient clinical evidence [19]. In addition to ZSS, SPI has been identified in a small number of other plants, including other Ziziphus species such as *Ziziphus mauritiana* Lam. (Rhamnaceae) [20], *Ziziphus spina-christi* (L.) Desf. (Rhamnaceae) [21], *Ziziphus jujuba* Mill. (Rhamnaceae) [22]. Notably, recent untargeted metabolomics research has further expanded the tissue distribution of SPI, reporting its presence in the fruit pulp of 15 Chinese jujube cultivars for the first time, along with 54 other characteristic polyphenols [23]. In addition to Chinese cultivars, SPI and its two bioactive ester derivatives (6′′′-hydroxybenzoylspinosin and 6′′′-feruloylspinosin) have also been detected in the seeds of Korean-grown *Ziziphus jujuba* cultivars (Mechu and Sanzoin), confirming the widespread distribution of SPI in this species across different geographical regions [24]. Other sources include *Strophioblachia fimbricalyx* Boerl. (Euphorbiaceae) [25], *Cayaponia tayuya* (Vell.) Cogn. (Cucurbitaceae) [3], *Passiflora edulis* Sims (Passifloraceae) [26]. *Leonurus japonicus* Houtt (Lamiaceae) [5], *Desmodium tortuosum* (Sw.) DC. (Leguminosae) [27], *Clutia abyssinica* Jaub. (Euphorbiaceae) [28], *Wilbrandia ebracteata* Cogn. (Cucurbitaceae) [29], *Acorus calamus* var. *angustatus* Besser (Acoraceae) [30]. However, SPI content in these plants is extremely low, and lacks ethnopharmacological evidence for sedative–hypnotic use, so they are not major sources for medicinal or pharmaceutical purposes. As a characteristic component of ZSS, SPI, together with jujuboside A and jujuboside B, is recognized as a critical Q-Marker of ZSS, and is the only component highly consistent with ZSS’s traditional sedative–hypnotic effect due to its good specificity, stability, and traceability [31]. The plant sources of SPI are summarized in Table 2.

## 4. Pharmacological Effects

### 4.1. Anti-Central Nervous System Diseases

**Figure 2:**
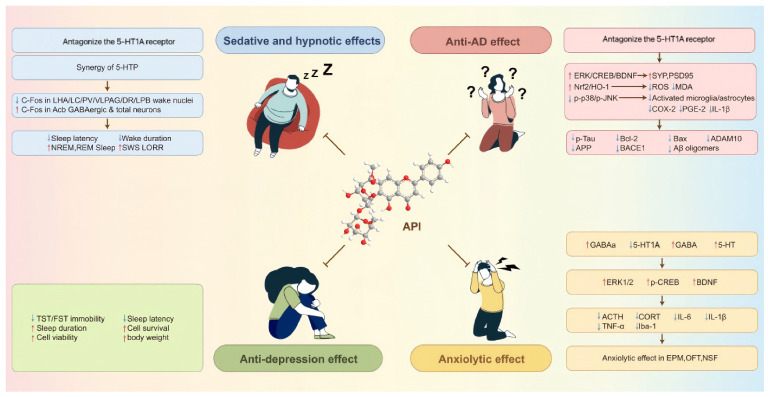
Mechanisms of protective action of SPI against central nervous system disease. ↑ means increase; ↓ means decrease.

Central nervous system (CNS) diseases represent a major global health burden, and conventional therapies have significant limitations [32]. SPI, a natural active compound, exerts multi-target therapeutic effects across various CNS disorders. This section systematically reviews SPI’s sedative–hypnotic, anti-Alzheimer’s, and anxiolytic/antidepressant activities and their underlying mechanisms (Figure 2).

#### 4.1.1. Sedative and Hypnotic Activity of SPI

The sleep–wake cycle in mammals consists of three distinct phases: wakefulness, non-rapid eye movement (NREM) sleep, and rapid eye movement (REM) sleep. Sleep disorders are essentially a category of diseases caused by dysregulation of the sleep–wake cycle [33]. Complex neuronal networks coordinately regulate the transition between sleep and wakefulness: activation of wakefulness-promoting neurons maintains the awake state, whereas activation of sleep-promoting neurons induces sleep. These neurons release various neurotransmitters that bind to corresponding specific receptors, ultimately generating characteristic electroencephalographic and electromyographic signals [34]. The occurrence of sleep disorders is closely associated with multiple factors, including abnormalities in sleep–wake neural circuits, imbalances in neurotransmitter systems, and abnormal receptor expression [35]. Therefore, it is of great significance to explore natural active ingredients that can safely modulate the sleep–wake pathway to improve sleep disorders. Among these natural products, SPI has attracted increasing attention for its remarkable sedative and hypnotic effects and high safety profile. Existing studies have shown that SPI exerts its sedative and hypnotic effects mainly by regulating the expression of neurotransmitters and hormones.

It is well-established that 5-Hydroxytryptamine (5-HT) plays a dual role in sleep–wake regulation: it promotes arousal and inhibits REM sleep during the daytime, acts as the precursor of melatonin at night, and modulates NREM sleep via the raphe nucleus- hypothalamus–cortex pathway and multiple receptor subtypes [36]. Consistent with the critical role of 5-HT in sleep homeostasis, L-tryptophan is catalyzed by tryptophan hydroxylase (TPH) to generate 5-HTP, which is the direct endogenous precursor for 5-HT biosynthesis. Studies have shown that SPI dose-dependently prolongs pentobarbital-induced sleep duration, shortens sleep latency, and increases the sleep-onset rate at subhypnotic pentobarbital doses in mice. Its hypnotic effect can be potentiated by 5-HTP; completely blocked by p-chlorophenylalanine (PCPA), a selective inhibitor of TPH, SPI can reverse the PCPA-induced reduction in sleep duration. In contrast, SPI exerts no significant effect on the reduction in pentobarbital-induced sleep duration caused by L-3-(3,4-dihydroxyphenyl) alanine (L-DOPA), the endogenous precursor of dopamine. These findings suggest that the sedative–hypnotic effect of SPI is closely associated with the enhancement of 5-HTergic neurotransmission and is not involved in modulating the dopaminergic system [37]. Beyond regulating 5-HT biosynthesis, mechanistic studies have identified the 5-hydroxytryptamine 1A (5-HT1A) receptor is the most widely distributed Gi/o protein-coupled 5-HT receptor subtype receptor as the core molecular target mediating SPI’s hypnotic effects. The 5-HT1A receptor is the most widely distributed Gi/o protein-coupled 5-HT receptor subtype in the central nervous system (CNS). It is classified into presynaptic autoreceptors and postsynaptic heteroreceptors, and serves as a key mediator of the 5-HTergic system in regulating the sleep–wake cycle. SPI exerts its pharmacological effects via dual antagonistic regulation of presynaptic and postsynaptic 5-HT1A receptors: it can not only antagonize postsynaptic 5-HT1A heteroreceptors and reverse the sleep-inhibitory effect mediated by its selective agonist 8-hydroxy-2-(di-n-propylamino)tetralin (8-OH-DPAT) [38], but also antagonize presynaptic 5-HT1A autoreceptors. Notably, SPI exhibits a significant synergistic hypnotic effect with p-MPPI. This classic selective 5-HT1A receptor antagonist provides direct pharmacological evidence that SPI and p-MPPI act on the same target pathway [39].

In addition to exerting its hypnotic effect by targeting the central 5-HT system, SPI also exerts a sedative–hypnotic effect via binding to central Gamma-Aminobutyric Acid (GABA) receptors. The results of competitive binding assays and molecular docking have confirmed that SPI can competitively bind to GABA receptors in the brain tissue of Sprague-Dawley (SD) rats with diazepam and exhibits favorable receptor binding activity, providing multi-target pharmacological support for its hypnotic effect [12].

Beyond the well-characterized molecular targets, recent studies have further elucidated the neural circuit mechanisms of SPI in sleep regulation, showing that it exerts a sedative–hypnotic effect by bidirectionally modulating antagonistic sleep–wake circuits. On one hand, SPI specifically inhibits the activity of wake-promoting orexin neurons in the lateral hypothalamic area (LHA), reduces c-Fos expression in this neuronal population, downregulates the hypothalamic expression of wake-promoting orexin-A, and concurrently upregulates sleep-promoting melanin-concentrating hormone (MCH) in sleep-deprived rats. This orexinergic inhibition further suppresses neuronal excitability in multiple downstream arousal-related brain regions, including the locus coeruleus (LC) [40]. On the other hand, it activates GABAergic sleep-promoting neurons in the nucleus accumbens (Acb) and increases c-Fos expression specifically in this neuronal subpopulation [41].

In conclusion, SPI exhibits notable sedative and hypnotic activity by modulating neurotransmitter systems, including serotonergic and GABAergic pathways, and by bidirectionally regulating sleep–wake neural circuits. Its multi-target mechanisms highlight its therapeutic potential for treating sleep disorders.

#### 4.1.2. Anti-Alzheimer’s Activity of SPI

Alzheimer’s disease (AD) is a chronic neurodegenerative disorder of the central nervous system with insidious onset, primarily affecting the elderly population. The core clinical manifestations of AD patients are progressive memory decline and cognitive dysfunction, which arise from three key pathological hallmarks: senile plaques formed by the deposition of β-amyloid peptide (Aβ) in the brain, neurofibrillary tangles (NFTs) induced by hyperphosphorylation of Tau protein (p-Tau), and synaptic dysfunction in neurons [42]. The pathogenesis of AD is highly intricate and multifactorial. In addition to the well-established Aβ and Tau hypotheses, AD is also closely associated with neuroinflammation, oxidative stress, cholinergic dysfunction, dysregulation of calcium homeostasis, impaired autophagy, and blood–brain barrier damage [43].

SPI improves learning and memory through the serotonergic system. Notably, the pro-cognitive effect of SPI is antagonized by a sub-effective dose of the 5-HT1A agonist 8-OH-DPAT, indicating that SPI acts functionally as a 5-HT1A antagonist rather than an agonist. Consistent with this, SPI increases the phosphorylation of Extracellular Signal-Regulated Kinase (ERK) and cAMP Response Element-Binding Protein (CREB) in the hippocampus, which represents a downstream consequence of SPI-augmented serotonergic signaling closely related to memory formation [44]. Notably, in addition to directly regulating the neurotransmitter system, SPI further enhances cognitive function by promoting neurogenesis. Studies have shown that it promotes neurogenesis in the hippocampal dentate gyrus, as reflected by increased neural cell proliferation and survival of, enhances generation of immature neurons, and leads to a higher proportion of newborn cells differentiating into mature neurons. At the molecular level, SPI significantly upregulates the expression of phosphorylated ERK, phosphorylated CREB, and mature Brain-Derived Neurotrophic Factor (BDNF) in the hippocampus, suggesting that it supports neurogenesis and cognitive improvement by activating the ERK/CREB/BDNF signaling pathway [45]. Beyond the aforementioned pro-cognitive mechanisms, SPI also exerts a remarkable inhibitory effect on AD-related neuroinflammation, a crucial neuroprotective pathway. Studies have demonstrated that SPI not only reduces the number of activated microglia and astrocytes induced by AβO injection, thus attenuating neuroinflammation at the source [46]. but also inhibits Aβ_25–35_-induced overexpression of cyclooxygenase-2 (COX-2) in acute hippocampal slice models, thereby decreasing the levels of downstream inflammatory factors, including PGE-2 and IL-1β, effectively relieving neuronal damage caused by neuroinflammation and exerting comprehensive neuroprotection [47].

Neuroinflammation and oxidative stress are closely interconnected and synergistically exacerbate the pathological progression of AD. Correspondingly, SPI exhibits distinct regulatory effects on oxidative stress and Aβ metabolism. It primarily suppresses the production of reactive oxygen species (ROS) by activating the nuclear factor erythroid 2-related factor 2 (Nrf2)/heme oxygenase-1 (HO-1) pathway. It simultaneously upregulates A Disintegrin And Metalloproteinase domain-containing protein 10 (ADAM10) expression while downregulating Beta-site APP Cleaving Enzyme 1 (BACE1) expression, thereby modulating the processing of amyloid precursor protein (APP) and reducing the generation of Aβ_1–42_ [48]. Meanwhile, SPI directly inhibits Aβ_1–42_ aggregation and lowers malondialdehyde (MDA) levels in the hippocampus, alleviating pathological protein burden and oxidative damage [49]. Furthermore, SPI reverses H_2_O_2_-induced excessive production of MDA and lactate dehydrogenase (LDH), Aβ secretion and aggregation, hyperphosphorylation of Tau protein, and synaptic dysfunction by inhibiting p38 activity, further strengthening its antioxidant and anti-pathological effects in synergy with the aforementioned anti-inflammatory mechanisms [50].

Synaptic plasticity dysfunction is a core pathological feature underlying cognitive decline in AD patients, and SPI also plays a vital role in preserving synaptic plasticity, a key step in cognitive improvement. Studies have confirmed that SPI effectively alleviates Aβ-induced impairment of long-term potentiation (LTP) and improves hippocampal synaptic function in 5XFAD transgenic AD mice by enhancing plasmin activity; this effect can be blocked by the plasmin inhibitor 6-aminocaproic acid, further verifying the mediating role of plasmin in this process [51]. Meanwhile, SPI reverses the AβO-induced decrease in choline acetyltransferase (ChAT) expression, indicating a protective effect on the cholinergic system. The stability of cholinergic function is essential for maintaining synaptic plasticity and cognitive performance, complementing the aforementioned synaptic-protective mechanisms [46].

In summary, SPI exerts comprehensive neuroprotective effects through multiple coordinated and complementary pathways, including neurotransmitter regulation, neurotrophic support, promotion of neurogenesis, anti-inflammatory action, antioxidative stress, and cholinergic protection. These findings provide a multi-target theoretical basis for the potential application of SPI in the treatment of Alzheimer’s disease and lay a foundation for further related research.

#### 4.1.3. Anti-Anxiety and Anti-Depression Activity of SPI

Mental disorders represented by anxiety and depression are closely related to the dysfunction of the CNS [52]. As a natural small-molecule compound, SPI has shown remarkable anti-anxiety and anti-depression effects in various animal models. The underlying mechanisms mainly involve regulating neurotransmitter levels, inhibiting oxidative stress and inflammatory response, and improving synaptic plasticity.

Studies have shown that SPI exerts a pivotal role in ameliorating anxiety disorders induced by chronic restraint stress (CRS). In CRS-induced anxious mice, oral administration of SPI (1.25, 2.5, and 5 mg/kg) ameliorated anxiety-like behaviors, mainly via regulating GABAA and 5-HT1A receptors. By activating these receptors, SPI further modulates the downstream ERK1/2/CREB/BDNF signaling pathway and significantly upregulates the protein expression of ERK1/2, p-CREB, and BDNF in the hippocampus (HPC) and prefrontal cortex (PFC). Such a cascade contributes to restoring disordered neurotransmitter levels (5-HT, GABA), attenuating the release of adrenocorticotropic hormone (ACTH), corticosterone (CORT), and inflammatory cytokines (interleukin-6 (IL-6), interleukin-1β (IL-1β) and tumor necrosis factor-α (TNF-α)), as well as suppressing microglial activation (downregulation of Iba-1). Ultimately, these molecular alterations led to significant anxiolytic effects in the elevated plus maze (EMP), open field test (OFT), and novelty suppressed feeding test (NSF) [53,54]. Furthermore, studies have shown that SPI exhibits antidepressant activity in both in vitro and in vivo models. In the chronic unpredictable mild stress (CUMS)-induced depression model in mice, the mixture of magnoflorine, SPI, and 6‴-feruloyspinosin (5, 10, and 20 μM) reduced immobility time in the TST and FST; concurrently, this mixture also promoted cell survival in the PC12 cell injury model, highlighting its neuroprotective potential [14].

SPI also demonstrates anti-post-traumatic stress disorder (PTSD) activities. PTSD is a severe mental disorder marked by increased arousal, intrusive symptoms, avoidance, and negative cognitive changes after exposure to traumatic stress [55]. Emerging evidence indicates that SPI exerts therapeutic effects against PTSD. In the single prolonged stress SPS-induced mouse model of PTSD, oral administration of SPI at 3 mg/kg significantly ameliorates PTSD-like behaviors, as validated by the elevated plus maze test, marble-burying test, Y-maze test, tail suspension test, and fear extinction test. Mechanistically, SPI facilitates fear extinction via antagonize of the 5-HT1A receptor. Consistently, SPI reverses the aberrant phosphorylation of protein kinase A (PKA) and CREB, the principal downstream signaling components of the 5-HT1A receptor, in the amygdala region of PTSD model mice. These findings suggest that SPI represents a promising natural therapeutic candidate for PTSD that may address the limitations of current pharmacotherapies [56].

### 4.2. Hepatoprotective Activity of SPI

**Figure 3:**
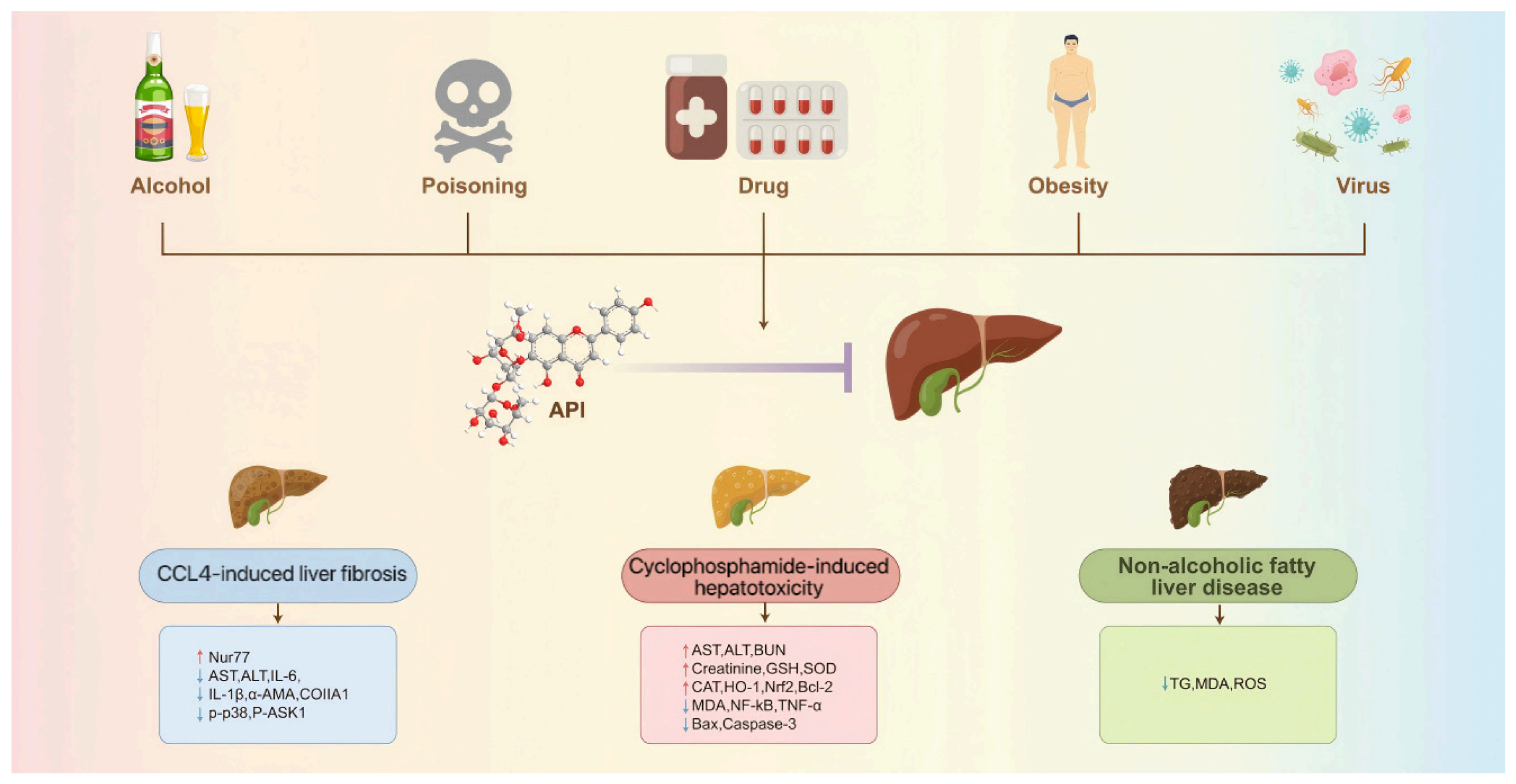
The hepatoprotective mechanism of SPI. ↑ means increase; ↓ means decrease.

In hepatic fibrosis, SPI can directly bind the ligand-binding domain of Nur77, upregulate Nur77 expression, and thereby block activation of the apoptosis signal-regulating kinase 1 (ASK1)/p38 mitogen-activated protein kinase (p38 MAPK) signaling pathway. It inhibits TGF-β1-induced activation of LX2 and HSC-T6 hepatic stellate cells in a dose-dependent manner, reduces collagen deposition and the release of inflammatory factors such as IL-6 and IL-1β, thereby improving CCl4-induced hepatic fibrosis in mice [57]. In the model of cyclophosphamide-induced hepatotoxicity and nephrotoxicity, SPI significantly reduces the levels of Aspartate Aminotransferase (AST), Alanine Aminotransferase (ALT), Blood Urea Nitrogen (BUN), and creatinine. It attenuates oxidative stress injury by upregulating the Nrf2/HO-1 pathway, increasing antioxidant indicators including Glutathione (GSH), Superoxide Dismutase (SOD), and Catalase (CAT), and decreasing MDA levels. Meanwhile, SPI suppresses Nuclear factor kappa-light-chain-enhancer of activated B cells (NF-κB)/TNF-α-mediated inflammation and regulates the B-cell lymphoma-2 (Bcl-2)/Bcl-2-associated X protein (Bax)/cysteine-aspartic acid protease-3 (Caspase-3) pathway to inhibit apoptosis, thus improving hepatic and renal function and histopathological damage [58]. SPI also exhibits protective effects against non-alcoholic fatty liver disease. In oleic acid-induced HepG2 cell models, it activates the peroxisome proliferator-activated receptor gamma coactivator-1α (PGC-1α) pathway, thereby alleviating intracellular lipid accumulation, reducing triglyceride levels, and decreasing reactive oxygen species and malondialdehyde production. These effects collectively promote the recovery from oleic acid-induced hepatic steatosis [59]. Collectively, SPI integrates anti-fibrotic, antioxidant, anti-inflammatory, and anti-apoptotic activities through coordinated regulation of Nur77/ASK1/p38 MAPK, Nrf2/HO-1, NF-κB, and apoptosis-related signaling pathways, highlighting its potential as a promising lead compound for the prevention and treatment of various liver diseases (Figure 3).

### 4.3. Antitumor Activity of SPI

SPI, a key active component derived from ZSS, has demonstrated significant anti-tumor potential in various tumor models. In studies on colorectal cancer, water-soluble polyphenols extracted from ZSS have been shown to inhibit cancer cell proliferation, induce apoptosis, and enhance the chemosensitivity of colorectal cancer cells. Animal experiments further indicate that this extract effectively suppresses the aberrant expression of early markers, including COX-2, EMR1, and Ki67 in a mouse model of colitis-associated colorectal cancer (CAC). Through purification with SP207 macroporous resin and analysis by reversed-phase high-performance liquid chromatography–tandem mass spectrometry (RP-HPLC-MS/MS), SPI was confirmed as the key active substance responsible for the anti-colorectal cancer activity of this water-soluble polyphenol extract [60]. Beyond colorectal cancer, SPI exhibits similar anti-tumor activity in breast cancer. Mechanistic studies suggest that SPI can reduce breast cancer cell viability and induce apoptosis by targeting and regulating AKT1 and Tumor Protein P53 (TP53) signaling pathways, indicating its potential for broad-spectrum inhibitory effects across different tumor types [61].

### 4.4. Bone Injury Protection Activity of SPI

Osteoarthritis (OA) is a prevalent chronic degenerative joint disorder defined by progressive articular cartilage degradation, subchondral bone destruction, and persistent chronic inflammatory damage. Excessive osteoclast activation, extracellular matrix loss, and oxidative stress are key pathological factors that promote OA progression and bone damage [62]. Recent studies have revealed that SPI exerts effective protective effects against bone and cartilage injury via multiple mechanisms. On the one hand, SPI can suppress EGFR-mediated AKT phosphorylation, thereby inhibiting nuclear factor of activated T cells c1 (Nfatc1)-mediated expression of osteoclast-related genes and the subsequent formation and function of osteoclasts. Through this mechanism, SPI effectively alleviates LPS-induced osteolysis, suggesting its potential as a promising candidate for preventing inflammatory osteolysis [63]. On the other hand, SPI displays significant chondroprotective effects. It attenuates tert-butyl hydroperoxide (TBHP)-induced degradation of the chondrocyte extracellular matrix. Further mechanistic studies demonstrate that SPI significantly activates the Nrf2/HO-1 signaling pathway and upregulates NADPH quinone dehydrogenase 1 (NQO1) in chondrocytes. Notably, silencing of Nrf2 by siRNA abolishes the cartilage-protective effects of SPI. Consistent with in vitro findings, SPI also exerts obvious ameliorative effects in mouse OA models [64].

Collectively, these findings indicate that SPI protects against bone and cartilage injury by inhibiting osteoclastogenesis through the EGFR/Akt/Nfatc1 pathway and ameliorating oxidative stress-induced chondrocyte dysfunction via activation of the Nrf2/HO-1 signaling pathway, highlighting its therapeutic potential for the treatment of osteoarthritis and inflammatory osteolysis.

### 4.5. Cardioprotective and Metabolotropic Activities

In addition, SPI exhibits favorable cardioprotective activity in the cardiovascular system. In a rat model of myocardial ischemia–reperfusion injury, pretreatment with SPI significantly alleviates myocardial tissue damage, reduces the release of serum myocardial enzymes, and inhibits cardiomyocyte apoptosis. Mechanistically, these protective effects are primarily mediated by inhibition of glycogen synthase kinase-3β (GSK3β) phosphorylation at Tyr216, which concurrently enhances autophagy and activates antioxidant signaling. Specifically, SPI enhances the protein expression levels of microtubule-associated protein light chain 3B-II (LC3B-II) and triggers the PGC-1α/Nrf2/HO-1 pathway, thereby strengthening cellular antioxidant capacity and ameliorating ischemia–reperfusion-induced myocardial injury [65]. Beyond acute myocardial injury, SPI also improves insulin resistance, dyslipidemia and vascular dysfunction in high-fat diet-fed mice and cultured vascular cells. It reduces oxidative stress and inflammation, enhances endothelial nitric oxide (NO) production, and exerts these vascular protective effects via regulating IRS-1 phosphorylation and activating the PI3K signaling pathway [66]. Overall, these findings show that SPI exerts multi-targeted cardioprotective effects by modulating autophagy, antioxidant and insulin signaling pathways, supporting its potential application in cardiovascular diseases.

**Figure 4:**
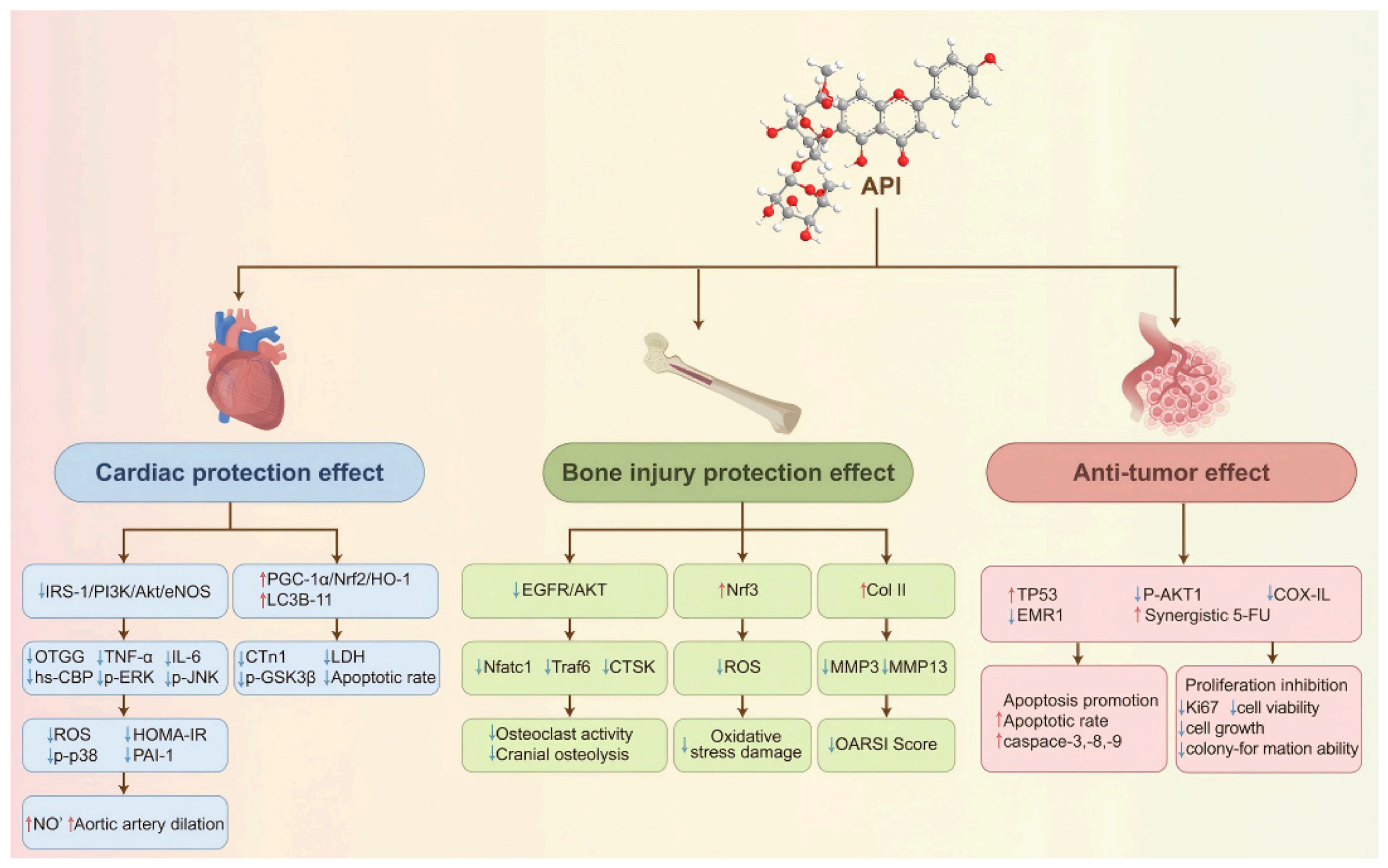
Mechanisms of other functions of SPI. ↑ means increase; ↓ means decrease.

The complete landscape of SPI’s pharmacological mechanisms across different systems is visualized in Figure 4. All the information regarding the pharmacological mechanism of SPI is summarized in Table 3.

## 5. Pharmacokinetics of SPI

**Figure 5:**
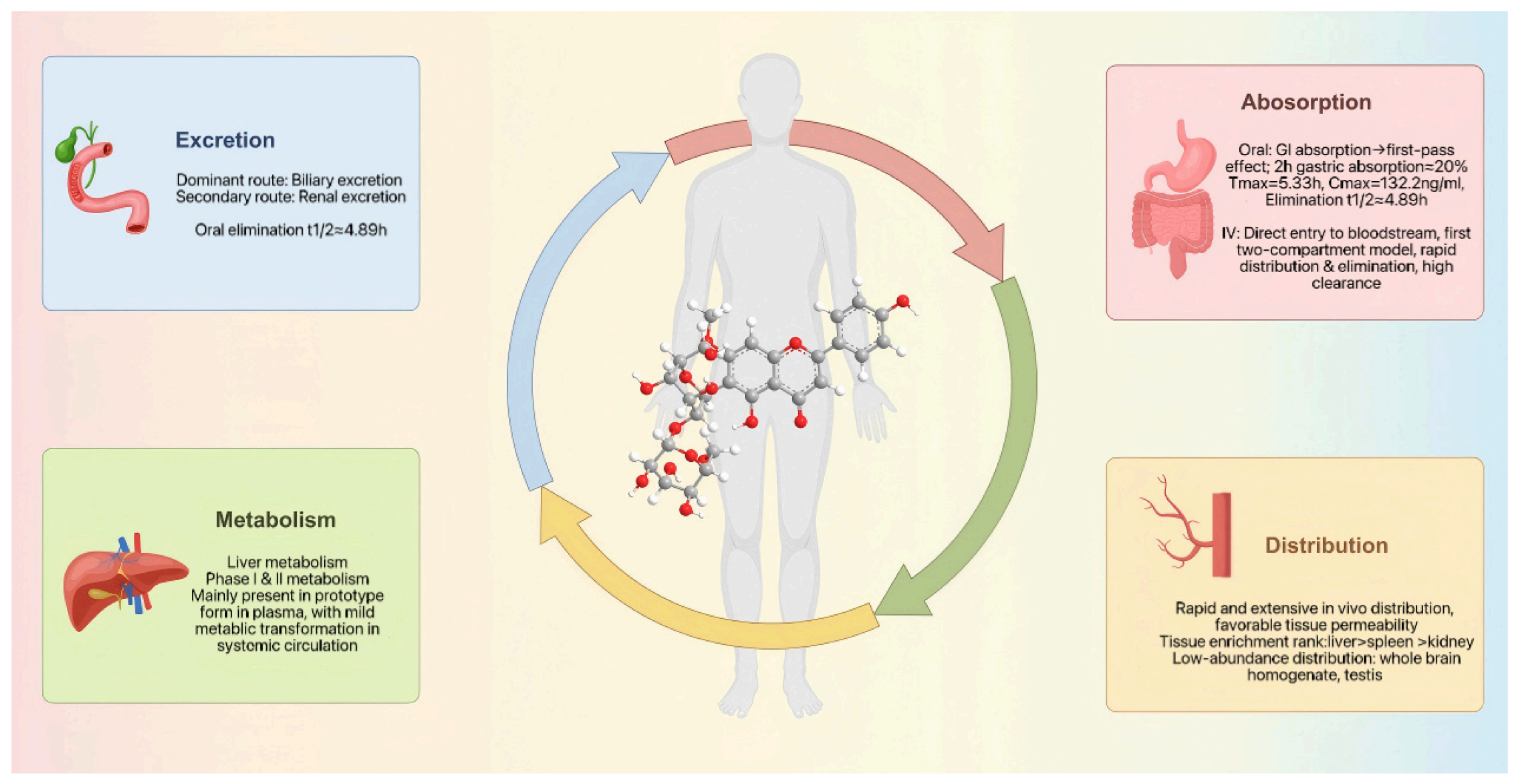
In vivo ADME characteristics of SPI.

The in vivo pharmacological efficacy of SPI is fundamentally determined by its absorption, distribution, metabolism, and excretion (ADME) process in the body, and systematic pharmacokinetic (PK) studies serve as the core bridge linking its in vitro bioactivity, in vivo therapeutic effect, and the ethnopharmacological application of ZSS. Over the past decades, extensive in vitro and in vivo studies have been conducted to decipher the PK behaviors of SPI under different administration routes, dosage forms, and compatibility conditions, as well as to explore the key factors governing its in vivo fate, which have deepened the scientific understanding of the traditional tranquilizing effect of ZSS and provided a core basis for the optimization of its clinical administration regimen (Figure 5).

In rat models, oral administration of SPI (20 mg/kg) resulted in a time to peak concentration (T_max_) of approximately 5.33 h, a peak concentration (C_max_) of 132.2 ng/mL, and an elimination half-life (t_1_/_2_) of approximately 4.89 h, with a low area under the concentration–time curve (AUC), indicating limited oral bioavailability [10], while after intravenous administration, the concentration–time curve of SPI conforms to a two-compartment model, characterized by rapid distribution and elimination, a high clearance rate, and a large apparent volume of distribution, suggesting that the compound can be rapidly and widely distributed in vivo. The aforementioned basic pharmacokinetic parameters lay a core foundation for the systematic elucidation of its complete ADME process [67]. Further in situ intestinal absorption studies have clarified the gastrointestinal absorption characteristics of SPI. The absorption percentages of SPI at different mass concentrations (10, 20, and 40 mg/L) in the rat stomach within 2 h were 20.35%, 21.88%, and 20.23%, respectively. SPI was absorbed in all segments of the rat gastrointestinal tract with an absorption process conforming to first-order kinetics and primarily driven by passive diffusion. Its intestinal absorption efficiency was not affected by drug concentration or intestinal pH within the tested range, and the absorption rate constant decreased in the following order: duodenum, colon, jejunum, and ileum, with favorable overall absorption in the stomach [68].

In terms of tissue distribution, SPI exhibits favorable tissue permeability. After intravenous administration, it rapidly accumulates in the liver, spleen, and kidney, with the highest concentration detected in the liver. In contrast, low average concentrations are found in whole brain homogenate and testis [67]. Subsequent brain region-specific studies have further refined these findings: SPI can effectively cross the BBB, and rather than being uniformly distributed throughout the brain, it is selectively enriched in specific regions, such as the striatum and hippocampus, which are directly associated with its central sedative and memory-improving pharmacological effects. This provides direct pharmacokinetic support for its central pharmacological activities [11]. In addition, 6′′′-feruloylspinosin, the main co-occurring flavonoid constituent structurally homologous to SPI in ZSS, can also be rapidly distributed and cross the BBB into brain tissue. Both compounds upregulate the expression of GABA receptor subtypes in hippocampal neurons, suggesting that multiple homologous flavonoids in ZSS exert synergistic central pharmacological activities [69].

The in vivo metabolic process of SPI has been systematically elucidated using a variety of modern analytical techniques. Studies have shown that after oral administration of ZSS flavonoid extract in rats, SPI and 6′′′-feruloylspinosin mainly exist in prototype form in plasma, with a T_max_ of 4–6 h after administration and slow in vivo elimination, suggesting that its overall metabolic transformation is relatively mild in the systemic circulation [70]. Although SPI primarily exists in its prototype form in plasma, in-depth metabolomic studies have supplemented that flavonoid C-glycosides from ZSS, including SPI, can undergo phase I and phase II metabolic reactions such as hydrogenation, hydrolysis, and glucuronidation in vivo, and dozens of related metabolites have been tentatively identified in rat body fluids and excreta via high-resolution mass spectrometry [71]. Notably, its metabolic kinetic characteristics are susceptible to regulation by TCM compatibility environments. In the classic TCM formula Zaoren Anshen Formula, the metabolic clearance rate of SPI is slowed, its in vivo action duration is significantly prolonged, and its oral bioavailability is further improved. In contrast, compatibility with Schisandrae Chinensis Fructus or Salviae Miltiorrhizae Radix et Rhizoma alone can accelerate its metabolic transformation, thereby reducing in vivo exposure. These findings fully demonstrate the complex regulatory effect of TCM compatibility on the metabolic fate of active constituents [72].

In terms of safety assessment for clinical combination medication, a key in vitro study confirmed that SPI has no significant inhibitory effect on 7 major human liver microsomal cytochrome P450 (CYP) enzyme subtypes, including CYP3A4 and CYP2D6 (all IC_50_ > 50 μmol/L), suggesting a low risk of causing metabolic drug–drug interactions via CYP inhibition [73]. This finding is further supported by preclinical pharmacokinetic evidence: no significant pharmacokinetic interaction was observed between SPI and donepezil (a first-line therapeutic drug for Alzheimer’s disease) in beagle dog models [74]. Notably, although SPI itself exhibits low CYP-inhibitory potential, its pharmacokinetic profile can be substantially altered when co-administered with other TCM herbs, as described above [72]. This apparent discrepancy suggests that the modulatory effects of TCM compatibility on the metabolic fate of SPI are mediated not by direct CYP inhibition but rather by alternative mechanisms, such as transporter modulation, enterohepatic circulation, or gut microbiota regulation, warranting further in-depth investigation.

The in vivo excretion of SPI and its metabolites is dominated by biliary excretion, supplemented by renal excretion. Existing studies have shown that SPI can be extensively excreted via the biliary route, a process precisely regulated by hepatic transporters. Part of the constituents excreted into the intestine via bile can be reabsorbed into the systemic circulation after hydrolysis by the gut microbiota, forming an enterohepatic circulation that prolongs their in vivo action. This characteristic is highly consistent with the traditional clinical application feature of ZSS, which is administered before bedtime to cover the nocturnal sleep cycle. Meanwhile, the proportion of prototype SPI in rat urine is extremely low, suggesting that the kidney is not the main excretion route for the prototype drug in rats, and its main form of renal excretion is phase II conjugated metabolites [71].

The in vivo disposition process of SPI is synergistically regulated by multiple factors, including transporter-mediated absorption and efflux, pathophysiological status, TCM compatibility, herbal matrix components, and traditional processing methods. SPI is a specific substrate of P-glycoprotein (P-gp) and multidrug resistance-associated proteins (MRPs), which are highly expressed in both intestinal epithelial cells and hepatocytes. Intestinal P-gp and MRPs efflux absorbed SPI back into the intestinal lumen, which is the core intrinsic mechanism underlying its low oral bioavailability. Hepatic P-gp further mediates its biliary excretion. Consistently, co-administration with the P-gp inhibitor cyclosporine A (CsA) significantly increases SPI exposure in blood, bile, and brain tissue, and markedly prolongs its half-life [17,75].

In addition, pathophysiological status can significantly alter the pharmacokinetic characteristics of SPI. In rat models of insomnia, the systemic exposure of SPI is decreased, and its clearance rate is accelerated. This finding provides modern scientific support for the traditional clinical experience of syndrome-based medication and dose adjustment of ZSS for patients with insomnia [76,77]. In addition to the regulation of compatibility on metabolism, herbal matrix components can also affect the in vivo absorption of SPI: long-term administration of polysaccharides from ZSS may inhibit the absorption of SPI by upregulating the expression of intestinal efflux proteins, revealing the complex bidirectional interaction between different matrix components and active constituents in medicinal herbs [78].

To overcome the core bottleneck of low oral bioavailability identified by pharmacokinetic studies, a variety of modern preparation strategies have been developed and validated. These novel formulations significantly improve the water solubility and oral absorption of SPI, and most are designed to align with the traditional bedtime administration regimen of ZSS. Detailed information on the development, characterization, and in vivo performance of these dosage forms is provided in the subsequent section.

## 6. Safety and Toxicological Profile

Existing studies have established that SPI exhibits an overall low-toxicity profile with an extremely wide safety window. Acute toxicity assessments have confirmed no lethal effects in rodents even at ultra-high doses up to 10 g/kg, which is consistent with the non-toxic characterization of ZSS in classic TCM literature [6].

Updated evidence further supplements its preclinical safety: For repeated-dose in vivo toxicity, 8-week continuous oral administration of SPI at 20 mg/kg/d in mice caused no abnormal changes in general behavior, body weight growth, hematological parameters, serum biochemistry, or histopathology of major vital organs (heart, liver, kidney, spleen and lung), filling the critical gap of medium-long term subchronic toxicity data of pure SPI monomer [64]. Subsequent 28-day repeated-dose studies further verified that SPI at doses up to 50 mg/kg/d did not induce hepatorenal toxicity in rodents, with all liver and kidney function markers within normal ranges and no pathological damage in target organs; notably, SPI also exerted a significant protective effect against drug-induced hepatorenal oxidative injury, which further confirmed its favorable organ safety profile while expanding its therapeutic application scenarios [58]. The latest 2026 study also confirmed that 14-day repeated administration of SPI at 10 times the clinical equivalent dose had no adverse effects on the growth status and organ health of mice, and its solid dispersion formulation developed for functional food applications shared the same good safety profile as the prototype SPI monomer [79].

In terms of cellular safety, updated in vitro studies clarified the upper safety concentration limit of SPI for central nervous system drug development: SPI had no cytotoxicity, proliferation inhibition or apoptosis-inducing effects on both normal and pathological model neuronal cells within its pharmacologically effective concentration range (0–100 μM); only at ultra-high concentrations exceeding 200 μM did it show slight proliferation inhibition on pathological model cells, while no toxic effect was observed on normal neuronal cells [50].

Collectively, existing preclinical evidence strongly supports that SPI exhibits an excellent safety profile with an extremely wide therapeutic window, consistent with the non-toxic characterization of ZSS in classic TCM literature. The available acute, subchronic, and cellular toxicity studies provide a solid preliminary safety basis for its development as a functional food ingredient and potential therapeutic agent.

However, several critical safety gaps remain unaddressed that must be resolved before clinical translation. First, no studies have evaluated the reproductive toxicity, genetic toxicity, or carcinogenicity of SPI, representing major unknowns for long-term human use. Second, all existing toxicological data are derived from healthy animal models, with no information available on the safety of SPI in special populations, including patients with hepatic or renal impairment, pregnant women, lactating women, and children. Third, while the prototype SPI monomer has demonstrated favorable safety, no systematic toxicological evaluations have been conducted for the advanced formulations (e.g., solid dispersions, phospholipid complex nanocarriers) developed to improve its bioavailability. These formulations may exhibit altered tissue distribution, accumulation, and clearance characteristics compared to free SPI, potentially leading to unforeseen organ toxicity. Finally, although in vitro studies have shown that SPI does not inhibit major human cytochrome P450 enzymes [73], there is a complete lack of in vivo drug–drug interaction studies in humans. The potential for SPI to interact with commonly used central nervous system drugs (e.g., benzodiazepines, antidepressants, antipsychotics) remains unknown, which is an important consideration for clinical combination therapy.

## 7. Druggability Optimization Strategies for SPI

Despite its broad-spectrum pharmacological activities and excellent safety profile, the clinical translation of SPI is severely limited by its poor druggability characteristics, including extremely low aqueous solubility, low oral bioavailability (<1%), and limited blood–brain barrier penetration. To overcome these bottlenecks, two main strategies have been explored in recent years: structural modification via natural derivatives and formulation optimization using novel drug delivery systems.

### 7.1. Natural Derivatives and Structure–Activity Relationships

**Figure 6:**
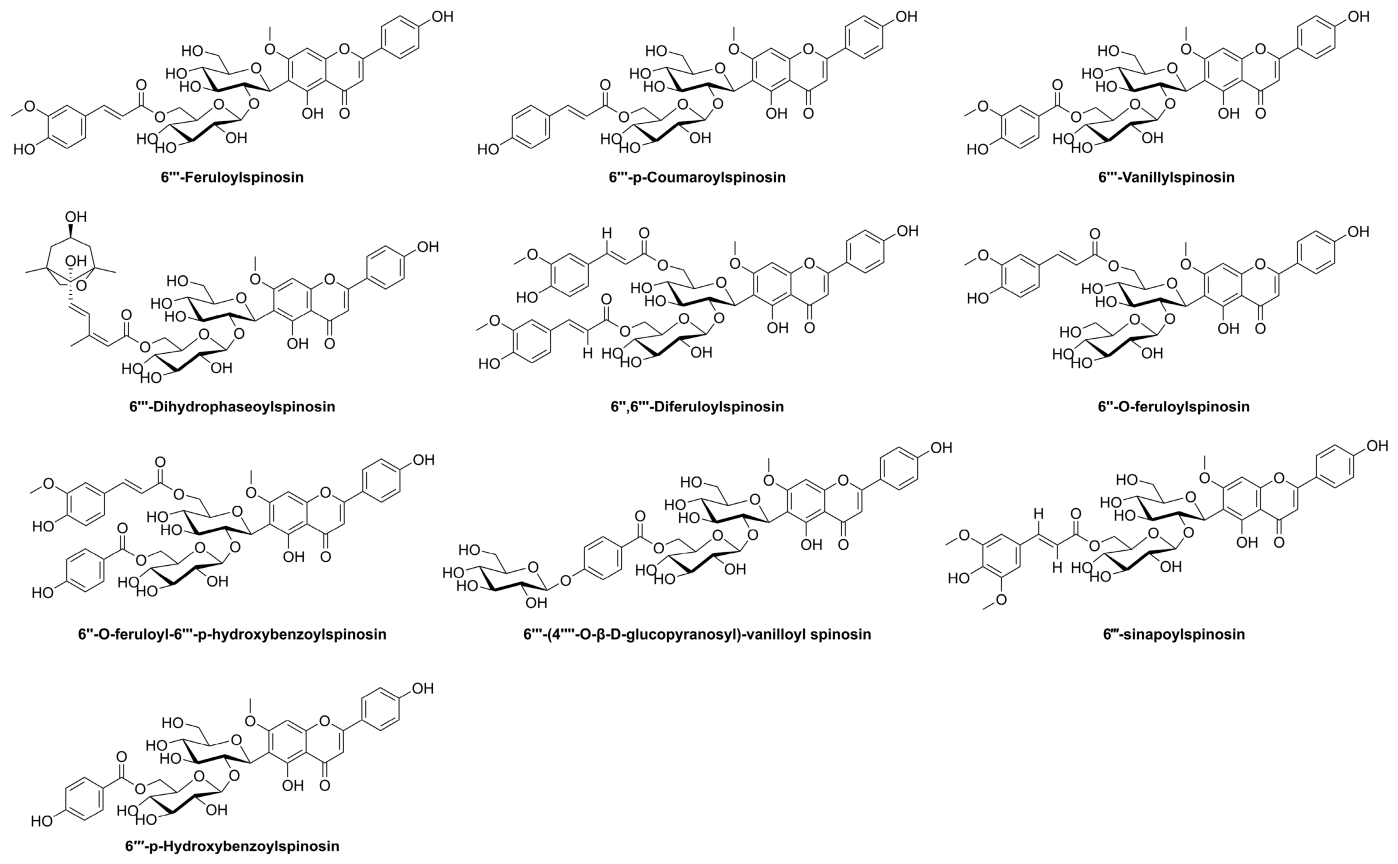
Structure diagrams of SPI derivatives.

Structural modification is a powerful approach to improve the pharmacokinetic properties and pharmacological activity of natural products. To date, more than 10 naturally occurring acylated derivatives of SPI have been isolated and identified from ZSS, most of which are formed by esterification of the 6′′′-hydroxyl group of SPI with different aromatic acids (Figure 6). These derivatives not only retain the core pharmacological activities of SPI but also exhibit improved lipophilicity and enhanced biological effects in some cases.

6′′′-Feruloylspinosin (6-FS) is a characteristic homologous flavonoid in ZSS with a content second only to SPI, and is also the most extensively studied natural derivative of SPI [18]. 6-FS exhibits rapid and extensive tissue distribution and is capable of crossing the blood–brain barrier. It can synergistically upregulate GABA receptor subtypes in hippocampal neurons with SPI, exerting sedative–hypnotic and neuroprotective effects, making it one of the core potential indicators for quality control of ZSS [69]. Studies have shown that 6-FS exerts ameliorative effects against pressure overload-induced heart failure complicated by insomnia via the activation of retinoid-related orphan receptor α (RORα). [80]. 6′′′-p-Coumaroylspinosin (P-CS) is a highly abundant homolog of SPI in ZSS with definite sedative–hypnotic activity, and constitutes the core pharmacodynamic material basis of flavonoids in ZSS together with SPI [81]. Studies have demonstrated that P-CS exhibits significant antioxidant activity. It can downregulate the abnormal expression of acrylamide-induced pro-apoptotic proteins Bax and Bim by inhibiting the JNKs signaling pathway, block excessive intracellular reactive oxygen species production, reverse glutathione depletion, and alleviate cellular oxidative stress disorders. This effect ultimately antagonizes oxidative stress-mediated neuronal apoptosis, providing core experimental evidence for its application as a therapeutic supplement in the prevention and management of neurodegenerative disorders [82]. 6′′′-Vanillylspinosin is a natural derivative isolated from ZSS, which retains the central sedative activity of SPI and serves as a key structural unit of diacylated derivatives [83].

In addition, 6′′′-dihydrophaseoylspinosin and 6″,6′′′-diferuloylspinosin, two representative acylated derivatives of SPI, were also isolated from the methanol extract of ZSS [18]. Other well-characterized naturally occurring acylated SPI derivatives include 6′′-O-feruloylspinosin and 6′′-O-feruloyl-6′′′-p-hydroxybenzoylspinosin, both of which have been reported to exert inhibitory effects on COX-1 and COX-2 enzymes [84]. Further reported natural SPI derivatives including 6‴-(4‴′-O-β-D-glucopyranosyl)-vanilloyl spinosin [83], as well as 6‴-sinapoylspinosin, and 6‴-p-hydroxybenzoylspinosin [85].

However, current research on SPI derivatives is still in its early stages. Most studies have focused on structural identification and preliminary activity screening, with limited investigation into their structure–activity relationships, pharmacokinetic profiles, and in vivo toxicity. The synergistic mechanisms between SPI and its derivatives remain poorly understood, hindering the development of standardized multi-component preparations.

### 7.2. Novel Drug Delivery Systems

Formulation optimization is the most widely used strategy to enhance the oral bioavailability of drugs with poor aqueous solubility. Various novel delivery systems have been developed for SPI, all of which aim to enhance its aqueous solubility and membrane permeability. The key advances in SPI formulation development are summarized below.

#### 7.2.1. Solid Dispersions

Solid dispersion technology is the most mature and industrially scalable approach for improving the solubility of poorly water-soluble drugs. When formulated into SD with hydrophilic polymers such as polyvinylpyrrolidone (PVP) and polyethylene glycol (PEG), SPI is converted into an amorphous state, significantly increasing its dissolution rate. Pharmacokinetic studies have shown that SPI-SD achieved a relative oral bioavailability of 219.61% compared with free SPI and significantly shortened the time to peak plasma concentration (tmax) [86].

To further improve the bioavailability, researchers have developed phospholipid complex-modified solid dispersions (SPI-PLC-SD). This formulation combines the advantages of phospholipid complexes (improved lipophilicity) and solid dispersions (improved aqueous solubility), resulting in a relative oral bioavailability of 265.39% compared with free SPI. Notably, SPI-PLC-SD also prolonged the tmax of SPI, indicating a sustained-release effect [87].

#### 7.2.2. Nanocarrier Delivery Systems

Nanocarriers have shown great potential for improving the oral bioavailability and brain targeting of natural products. Several nanocarrier systems have been developed for SPI, including solid lipid nanoparticles (SLNs) and self-microemulsifying drug delivery systems (SMEDDS).

Building on this work, researchers further combined PLC technology with nanocarrier delivery systems to fabricate SPI-PLC-loaded solid lipid nanoparticles (SPI-PLC-SLNs), where SPI exists in an amorphous form within the PLC. The optimized SPI-PLC-SLNs had an encapsulation efficiency of (82.91 ± 0.83)%, drug loading of (4.91 ± 0.25)%, average particle size of (193.12 ± 5.84) nm, polydispersity index (PDI) of 0.202 ± 0.055, and zeta potential of (−9.6 ± 1.8) mV. Compared with free SPI and SPI-PLC, SPI-PLC-SLNs markedly accelerated SPI dissolution; pharmacokinetic results showed SPI-PLC had a 2.02-fold higher relative oral bioavailability than free SPI, while SPI-PLC-SLNs further increased this value to 3.78-fold that of crude SPI. Furthermore, an SPI-PLC-based self-microemulsifying drug delivery system (SPI-PLC-SMEDDS) with uniform nanoscale particle size and excellent in vitro dispersibility was developed. Caco-2 cell monolayer studies confirmed that SPI-PLC-SMEDDS significantly enhanced SPI’s cellular uptake and transepithelial transport, and in vivo pharmacokinetic experiments demonstrated its oral bioavailability was statistically significantly higher than free SPI, reaching nearly 5-fold that of crude SPI, with additional immunomodulatory activity observed for this formulation [9].

Overall, current studies on novel SPI formulations primarily focus on improving its aqueous solubility and oral bioavailability via oral delivery systems, nearly all of which are limited to laboratory-based preclinical screening. The core bottleneck for clinical translation is the severe lack of systematic safety evaluation of these formulations, with no dedicated toxicological studies reported to date. Meanwhile, major research gaps remain in CNS-targeted delivery systems (including BBB penetration and nose-to-brain delivery) aligned with SPI’s core pharmacology, as well as SPI nanocrystals/nanosuspensions with excellent industrialization potential. Future research should prioritize systematic preclinical safety assessment and focus on CNS-targeted delivery and clinically scalable formulation technologies to advance the clinical translation of SPI.

## 8. Conclusions and Future Perspectives

This review provides the most comprehensive and up-to-date synthesis of SPI research to date, covering all relevant literature published up to 31 May 2026. By integrating evidence from phytochemistry, pharmacology, pharmacokinetics, toxicology, and pharmaceutical development, we have identified SPI as a unique molecular bridge between the millennia-old ethnopharmacology of ZSS and modern neuropharmacology. The compiled evidence not only validates the traditional use of ZSS for insomnia and anxiety but also reveals its broader therapeutic potential for neurological, hepatic, and cardiovascular disorders. A particularly significant finding is the remarkable alignment between SPI’s pharmacological profile and the classical TCM principles of “calming the mind” and “nourishing the liver”: at the molecular level, SPI exerts its sedative–hypnotic effect through a dual mechanism that simultaneously inhibits wake-promoting orexin neurons in the lateral hypothalamus and activates sleep-promoting GABAergic neurons in the nucleus accumbens, while modulating serotonergic neurotransmission via 5-HT_1_A receptors. This multi-target mechanism explains why ZSS has been clinically effective for centuries without causing the severe side effects and dependence associated with conventional benzodiazepine drugs. Furthermore, we have resolved a long-standing paradox in TCM pharmacology: while SPI as a pure monomer exhibits oral bioavailability below 1%, ZSS decoctions and processed forms remain clinically effective due to two scientifically validated traditional practices—stir-frying, which disrupts the seed coat to enhance aqueous dissolution of SPI, and formula compatibility, which prolongs systemic exposure through efflux transporter modulation and enterohepatic circulation—underscoring that the therapeutic value of TCM is not merely molecular but also pharmaceutical and context-dependent.

Notwithstanding these advances, the current evidence warrants cautious interpretation. Most animal studies are limited by small sample sizes and insufficient reporting of randomization and blinding, increasing the risk of bias. The reproducibility of key findings is largely unverified, as they mostly originate from single laboratories and are confounded by heterogeneity in SPI purity, dosing, and animal models. Much of the mechanistic evidence relies on in vitro systems using supraphysiological concentrations that exceed achievable brain exposure and cannot recapitulate metabolism or blood–brain barrier transport. Moreover, inconsistencies remain among published findings—particularly regarding dose–response relationships and the relative contribution of parent compound versus metabolites—that have yet to be reconciled. Current conclusions on SPI’s efficacy and mechanisms should therefore be regarded as preliminary.

Despite these promising advances, several critical bottlenecks must be addressed before SPI can be translated into clinical practice. All existing pharmacokinetic and efficacy data are derived from animal models, with a complete absence of human clinical trials—no first-in-human pharmacokinetic studies or proof-of-concept clinical trials having been published to date—representing the single most significant barrier to clinical development. While novel formulations have improved oral bioavailability by up to 5-fold, the limited blood–brain barrier penetration of SPI remains unresolved, and no dedicated brain-targeted delivery systems have been developed. The safety profile of SPI, although favorable in preclinical studies, is incomplete: reproductive toxicity, genetic toxicity, and long-term carcinogenicity data are completely absent, and no systematic safety evaluations have been conducted for advanced formulations. Additionally, the relative contribution of SPI metabolites and the gut microbiota–brain axis to its in vivo efficacy remains unknown. Beyond these scientific gaps, key translational bottlenecks persist: SPI must navigate complex regulatory pathways for botanical drugs, requiring standardized reference materials and validated quality control; the scalability of laboratory-scale formulations such as nanocarriers to industrial production—including batch reproducibility and stability—remains untested; and the low extraction yield and complex purification of high-purity SPI raise concerns about manufacturing feasibility and cost-effectiveness at commercial scale.

Based on these findings, future SPI research should prioritize five interconnected directions. First, establish a structured clinical development pathway progressing from first-in-human pharmacokinetic studies in healthy volunteers to phase II proof-of-concept trials for primary insomnia and mild cognitive impairment. Second, develop and evaluate nose-to-brain delivery systems and receptor-mediated BBB-penetrating nanocarriers to overcome the limited brain exposure of SPI, while addressing formulation scalability and reproducible large-scale production. Third, perform systematic reproductive toxicity, genetic toxicity, and subchronic toxicity studies of both SPI monomer and its advanced formulations to support clinical translation and regulatory submission. Fourth, investigate the pharmacological activity of SPI metabolites and the role of gut microbiota in mediating its central effects using metabolomics and microbiome profiling. Finally, explore the synergistic effects between SPI and its natural derivatives, such as 6′′′-feruloylspinosin, to develop standardized multi-component preparations that retain the holistic benefits of ZSS while meeting modern quality standards. Throughout, future preclinical studies should adopt rigorous methodological standards—including randomization, blinding, adequate powering, and independent replication—to enhance reproducibility.

SPI represents a paradigm for the modernization of TCM: a natural product with a long history of safe human use, well-characterized pharmacological mechanisms, and clear translational potential. By strategically addressing the identified bottlenecks, SPI has a tangible path to become a clinically versatile neuroactive agent that combines the wisdom of traditional medicine with the rigor of modern pharmaceutical science. This review provides a comprehensive roadmap for future research and development, aiming to accelerate the translation of this promising natural compound from the laboratory to the clinic.

## Figures and Tables

**Table 1:** Physical and chemical properties of SPI.

Name	Spinosin
Alias	2′′-beta-o-glucopyranosyl swertisin
Source	*Ziziphus jujuba* Mill. var. *spinosa* (Bunge) Hu ex H.F. Chou (Ziziphi Spinosae Semen)
CAS No	72063-39-9
Molecular formula	C_28_H_32_O_15_
Molecular weight	608.55 g/mol
Form	Powder
Color	Pale yellow to light yellow
Density	1.72 g/cm^3^ (Predicted)
Melting point	149 °C
Refractive index	1.733
Solubility	DMSO: slightly soluble; Methanol: slightly soluble; Water: slightly soluble
Polar surface area (PSA)	294.2 Å^2^
Log *p*	−1.07
Molar refractivity	143.46
Storage conditions	Store sealed, away from moisture and light, refrigerated (2–8 °C)

**Table 2:** The main plant source of SPI.

Plant Specials	Family	Used Part	Extract	Ref.
*Ziziphus jujuba* Mill. var. *spinosa*	Rhamnaceae	Dried ripe seed	70% ethanol reflux extraction	[19]
*Ziziphus mauritiana* Lam.	Rhamnaceae	Dried ripe seed	95% ethanol reflux extraction	[20]
*Ziziphus spina-christi* (L.) Desf.	Rhamnaceae	Dried ripe seed	80% methanol maceration	[21]
*Ziziphus jujuba* Mill.	Rhamnaceae	Dried ripe seed	70% methanol-formic acid ultrasonic extraction	[22]
*Strophioblachia fimbricalyx* Boerl.	Euphorbiaceae	Aerial portions	Methanol maceration	[25]
*Cayaponia tayuya* (Vell.) Cogn.	Cucurbitaceae	Dried roots	Chloroform exhaustive extraction; Methanol exhaustive extraction, liquid–liquid partition with butanol	[3]
*Passiflora edulis* Sims	Passifloraceae	Fresh pericarp	Aqueous infusion, n-butanol partition	[26]
*Leonurus japonicus* Houtt.	Lamiaceae	Aerial portions	95% ethanol maceration extraction, n-butanol partition	[5]
*Desmodium tortuosum* (Sw.) DC.	Leguminosae	Aerial portions	Methanol exhaustive extraction	[27]
*Clutia abyssinica* Jaub.	Euphorbiaceae	Leaves	Petroleum ether, chloroform and methanol cold percolation	[28]
*Wilbrandia ebracteata* Cogn.	Cucurbitaceae	Fresh crushed roots	Ethanol maceration	[29]
*Acorus calamus* var. *angustatus* Besser	Acoraceae	Rhizome	-	[30]

**Table 3:** (**a**) Pharmacological mechanisms of SPI on the CNS. (**b**) Pharmacological mechanisms of SPI on peripheral systems.

**(a)**					
**Pharmacological Effects**	**Experimental Model**	**Dosage/Concentration**	**Major Pharmacological Outcome**	**Underlying Mechanisms**	**Ref.**
Sedative and hypnotic effects	Pentobarbital-induced insomnia in mice (in vivo)	0.1 mL/10 g	Prolonged sleep time; shortened sleep latency; increased sleep-onset rate	Enhanced 5-HTergic neurotransmission; synergistic effect with 5-HTP	[37]
	Pentobarbital-induced insomnia rats (in vivo)	15 mg/kg	Increased NREM, SWS and REM sleep time; shortened sleep latency	Antagonism of postsynaptic 5-HT1A receptors	[38]
	Pentobarbital-induced insomnia mice (in vivo)	15 mg/kg	Increased LORR rate; attenuated 8-OH-DPAT-induced hypothermia	Antagonism of 5-HT1A receptors	[39]
	Naive mice (in vivo)	15 mg/kg	Reduced arousal-related neuronal activity	Inhibited c-Fos expression in LHA orexin neurons and LC	[40]
Anti-AD effect	Scopolamine-induced memory impairment mice (in vivo)	10, 20 mg/kg	Ameliorated memory deficits	Upregulated ERK and CREB phosphorylation; pro-cognitive effect blocked by 5-HT1A agonist	[44]
	AβO-induced memory impairment mice (in vivo)	20 mg/kg	Improved cognitive function	Increased ChAT level; inhibited microglia/astrocyte activation and Aβ_1–42_ oligomer formation	[46]
	Naive mice (in vivo)	5 mg/kg	Promoted hippocampal neurogenesis	Upregulated ERK, CREB and BDNF levels; increased proliferation/survival of neuronal cells and number of immature neurons in hippocampal dentate gyrus	[45]
	Aβ_1–42_-induced AD mice (in vivo)	100 μg/kg	Ameliorated AD pathological damage and cognitive impairment	Upregulated BDNF and Bcl-2 levels; downregulated MDA, Aβ_1–42_ and IL-6 levels	[49]
	5XFAD mice (in vivo)	30 μM	Improved synaptic plasticity	Upregulated plasmin level; enhanced LTP	[51]
	N2a/APP695 (in vitro)	25 μM	Inhibited Aβ production and oxidative stress	Upregulated Nrf2, HO-1 and ADAM10 levels; downregulated Aβ_1–42_, ROS, APP and BACE1 levels	[48]
	N2a/APP695 (in vitro)	25 μM	Improved synaptic function; attenuated Tau hyperphosphorylation	Upregulated synaptic proteins SYP and PSD95; downregulated phosphorylation of Tau, p38 and JNK	[50]
	Aβ_25–35_-injured mouse hippocampal slices (in vitro)	30 μmol/L	Improved synaptic plasticity; reduced neuronal apoptosis and neuroinflammation	Enhanced LTP; downregulated Aβ_25–35_, IL-1β, PGE-2, COX-2 and Bax levels	[47]
Anxiolytic effect	Male ICR mice (in vivo)	1.25, 2.5, 5 mg/kg	Exerted anxiolytic effect in elevated plus maze (EPM), open-field test (OFT) and light/dark box test	Modulated neurotransmitter receptors: downregulated 5-HT1A, upregulated GABAA	[53]
	CRS-induced anxious mice (in vivo)	1.25, 2.5, 5 mg/kg	Exerted anxiolytic effect in EPM, OFT and novelty suppressed feeding test (NSF)	Upregulated ERK1/2, p-CREB, BDNF, 5-HT and GABA levels; downregulated ACTH, CORT, pro-inflammatory cytokines (IL-6, IL-1β, TNF-α) and Iba-1	[54]
Anti-depressant effect	CUMS-induced depressed mice (in vivo)/PC12 (in vitro)	Mixture of magnoflorine, spinosin, and 6‴-feruloyspinosin 5, 10, 20 μM	Exerted antidepressant-like effect; improved PC12 cell survival	Decreased immobility time in tail suspension test (TST) and forced swimming test (FST) (in vivo); increased cell survival (in vitro)	[14]
Anti-PTSD effect	SPS-induced PTSD mice (in vivo)	3 mg/kg	Ameliorated PTSD-like behaviors: increased open arm time, memory extinction and spontaneous alternation; decreased buried marbles number, immobility time and fear response	Modulated 5-HT_1_A receptor and downstream PKA/CREB signaling: downregulated 5-HT_1_A, p-PKA and p-CREB levels	[56]
**(b)**					
**Pharmacological Effects**	**Experimental Model**	**Dosage/Concentration**	**Major Pharmacological Outcome**	**Underlying Mechanisms**	**Ref.**
Anti-tumor effect	AOM/DSS-induced colitis-associated cancer mice (in vivo)/HCT-116, HCT-8, HCT-8FU cells (in vitro)	ZSSP: 0–400 μg/kg (in vivo), 0–200 μg/mL (in vitro); SPI-Na^+^: 0–250 μg/mL (in vitro)	Inhibited colonic tumor growth; prolonged colon length (in vivo); suppressed tumor cell proliferation and colony formation (in vitro)	Upregulated apoptotic rate, activated caspase-3/8/9 and 5-FU chemosensitivity; downregulated cell growth, colony-formation ability, Ki67, EMR1 and COX-II	[60]
	MCF-7 cells (in vitro)	6.25, 12.5, 25, 50, 100 μg/mL	Inhibited breast cancer cell viability; induced cell apoptosis	Upregulated AKT1 and TP53 expression; increased apoptosis rate	[61]
Bone injury protection effect	RANKL/LPS-induced osteoclasts from BMMs (in vitro)	400 μM	Inhibited osteoclast activity and cranial osteolysis	Downregulated CTSK, Nfatc1, Traf6, EGFR and AKT expression	[63]
	DMM-induced osteoarthritis (OA) mice (in vivo)/C28/I2 chondrocytes (in vitro)	20 mg/kg (in vivo)/25 μM (in vitro)	Ameliorated OA progression; attenuated TBHP-induced chondrocyte damage and oxidative stress	Upregulated Nrf2 and type II collagen (Col II) levels; downregulated ROS, MMP3, MMP13 and OARSI score	[64]
Hepatoprotective effect	CCl_4_-induced liver fibrosis mice (in vivo)/LX2, HSC-T6 cells (in vitro)	20, 40 mg/kg (in vivo)/10, 20 μM (in vitro)	Attenuated liver fibrosis and hepatic function injury	Upregulated Nur77 expression; downregulated ALT, AST, pro-inflammatory cytokines (IL-6, IL-1β), α-SMA, Col1A1, p-p38 and p-ASK1	[57]
	CYC-induced hepatorenal toxicity mice (in vivo)	5, 10, 20 mg/kg	Alleviated hepatorenal function damage; reduced oxidative stress and cell apoptosis	Upregulated antioxidant enzymes (GSH, SOD, CAT), HO-1, Nrf2 and Bcl-2; downregulated AST, ALT, BUN, MDA, NF-κB, TNF-α, Bax and caspase-3	[58]
	OA-induced HepG2 cells (in vitro)	5, 10, 20 μM	Ameliorated hepatic steatosis and oxidative stress	Downregulated triglyceride (TG), MDA and ROS levels	[59]
Cardiovascular protection effect	LAD-induced myocardial ischemia mice (in vivo)	5 mg/kg	Attenuated myocardial ischemia injury; reduced cardiomyocyte apoptosis	Promoted cell autophagy; upregulated LC3B-II, PGC-1α, Nrf2 and HO-1; downregulated cTnI, LDH, apoptosis rate and p-GSK3β	[65]
	HFD-induced insulin resistance (IR) mice (in vivo)/HUVEC cells (in vitro)	20 mg/kg (in vivo)/20 μM (in vitro)	Improved glycolipid metabolism and insulin resistance; ameliorated vascular endothelial dysfunction	Downregulated body weight, fasting glucose, insulin resistance index, serum lipid, ROS, pro-inflammatory factors (TNF-α, IL-6), PAI-1, hs-CRP, sICAM-1, phosphorylation of p38/ERK/JNK/IRS-1 and p-eNOS; upregulated NO level	[66]

Abbreviations: 5-HT, 5-hydroxytryptamine; 5-HT1A, 5-hydroxytryptamine 1A; 8-OH-DPAT, 8-hydroxy-2-(di-n-propylamino)tetralin; Aβ, amyloid-β; ChAT, choline acetyltransferase; CREB, cAMP response element-binding protein; ERK, extracellular signal-regulated kinase; LC, locus coeruleus; LHA, lateral hypothalamic area; LORR, loss of righting reflex; NREM, non-rapid eye movement; REM, rapid eye movement; SWS, slow-wave sleep. Aβ, amyloid-β; ACTH, adrenocorticotropic hormone; AD, Alzheimer’s disease; BACE1, β-site amyloid precursor protein cleaving enzyme 1; BDNF, brain-derived neurotrophic factor; Bcl-2, B-cell lymphoma 2; ChAT, choline acetyltransferase; CORT, corticosterone; COX-2, cyclooxygenase-2; CREB, cAMP response element-binding protein; CRS, chronic restraint stress; CUMS, chronic unpredictable mild stress; EPM, elevated plus maze; ERK, extracellular signal-regulated kinase; GABA_A, γ-aminobutyric acid A; HO-1, heme oxygenase-1; IL, interleukin; JNK, c-Jun N-terminal kinase; LTP, long-term potentiation; MDA, malondialdehyde; Nrf2, nuclear factor erythroid 2-related factor 2; NSF, novelty suppressed feeding test; OFT, open-field test; PGE-2, prostaglandin E2; PKA, protein kinase A; PSD95, postsynaptic density protein 95; PTSD, post-traumatic stress disorder; ROS, reactive oxygen species; SPS, single prolonged stress; SYP, synaptophysin; TNF-α, tumor necrosis factor-α; TST, tail suspension test; FST, forced swimming test; AOM/DSS, azoxymethane/dextran sulfate sodium; ALT, alanine aminotransferase; AST, aspartate aminotransferase; BMMs, bone marrow-derived macrophages; BUN, blood urea nitrogen; CAT, catalase; Col II, type II collagen; COX-II, cyclooxygenase-II; CTSK, cathepsin K; CYC, cyclophosphamide; DMM, destabilization of the medial meniscus; GSH, glutathione; HFD, high-fat diet; HUVEC, human umbilical vein endothelial cells; LAD, left anterior descending coronary artery ligation; LDH, lactate dehydrogenase; MMP, matrix metalloproteinase; NO, nitric oxide; OA, osteoarthritis; OARSI, Osteoarthritis Research Society International; PAI-1, plasminogen activator inhibitor-1; PGC-1α, peroxisome proliferator-activated receptor gamma coactivator 1-alpha; SOD, superoxide dismutase; cTnI, cardiac troponin I.

## Data Availability

No new data were created or analyzed in this study. Data sharing is not applicable to this article.

## Abbreviations

The following abbreviations are used in this manuscript:

ZSS	Ziziphi Spinosae Semen
TCM	traditional Chinese medicine
MPLC	medium-pressure liquid chromatography
HPLC	high-performance liquid chromatography
NREM	non-rapid eye movement
5-HT	5-hydroxytryptamine
CNS	central nervous system
LHA	lateral hypothalamic area
LC	locus coeruleus
AD	Alzheimer’s disease
ERK	extracellular signal-regulated kinase
BDNF	brain-derived neurotrophic factor
Nrf2	nuclear factor-erythroid 2-related factor 2
ADAM10	A Disintegrin And Metalloproteinase domain-containing protein 10
APP	amyloid precursor protein
LDH	lactate dehydrogenase
ChAT	choline acetyltransferase
GABA	γ-Aminobutyric acid
PFC	prefrontal cortex
ASK1	Apoptosis signal-regulating kinase 1
AST	Aspartate Aminotransferase
BUN	Blood Urea Nitrogen
SOD	Superoxide Dismutase
NF-κB	nuclear factor kappa-light-chain-enhancer of activated B cells
OA	Osteoarthritis
NQO1	NADPH quinone dehydrogenase 1
PKA	Protein kinase A
LC3B-II	light chain 3B-II
P-gp	P-glycoprotein
CsA	cyclosporine A
6-FS	6′′′-Feruloylspinosin
P-CS	6′′′-p-Coumaroylspinosin
AUC	area under the concentration–time curve
SPI	spinosin
BBB	blood–brain barrier
HSCCC	high-speed countercurrent chromatography
NMR	nuclear magnetic resonance
REM	rapid eye movement
TPH	tryptophan hydroxylase
SD	Sprague-Dawley
MCH	melanin-concentrating hormone
Acb	accumbens
NFTs	neurofibrillary tangles
CREB	cAMP-response element binding protein
ROS	reactive oxygen species
HO-1	heme oxygenase-1
BACE1	Beta-site APP Cleaving Enzyme 1
MDA	malondialdehyde
LTP	long-term potentiation
CRS	chronic restraint stress
HPC	hippocampus
PGC-1α	Peroxisome proliferator-activated receptor gamma coactivator 1-alpha
p38 MAPK	p38 Mitogen-Activated Protein Kinase
ALT	Alanine Aminotransferase
GSH	Glutathione
CAT	Catalase
TP53	Tumor Protein P53
PTSD	Post-traumatic stress disorder
Nfatc1	nuclear factor of activated T cells c1
GSK3β	glycogen synthase kinase-3β
PK	pharmacokinetic
MRPs	multidrug resistance-associated proteins
CYP	cytochrome P450
RORα	retinoid-related orphan receptor α
SD	Solid dispersion
Cmax	peak plasma concentration
